# Supplementary material for: Integrated molecular and functional characterization of the intrinsic apoptotic machinery identifies therapeutic vulnerabilities in glioma
Source: Nat Commun. 2024 Nov 21;15:10089. doi: 10.1038/s41467-024-54138-9 (PMC11582606; doi:10.1038/s41467-024-54138-9)
Supplement: Supplementary file 1 — Supplementary Information [file 41467_2024_54138_MOESM1_ESM.pdf]

Supplemental Table 1: Patient Characteristics

Patient Tumours	Final Diagnosis	Tumour Status	Recurrence Number	Lobe	Sex
PT144	Glioblastoma, IDHwt, WHO Grade 4	Recurrent	3	Left Temporal	F
PT149	Astrocytoma, IDHmt, WHO Grade 3	Primary	0	N/A	F
PT163	Astrocytoma, IDHmt, WHO Grade 2	Primary	0	Right Temporal	F
PT201	Oligodendroglioma, IDHmt, 1p19q_codel, WHO Grade 2	Primary	0	Both Frontal	M
PT202	Oligodendroglioma, IDHmt, 1p19q_codel, WHO Grade 2	Primary	0	Left Frontal	F
PT211	Glioblastoma, IDHwt, WHO Grade 4	Recurrent	2	Right Parietal	F
PT225	Glioblastoma, IDHwt, WHO Grade 4	Recurrent	2	Left Frontal	F
PT239	Glioblastoma, IDHwt, WHO Grade 4	Recurrent	1	Right Temporal	M
PT244	Glioblastoma, IDHwt, WHO Grade 4	Recurrent	3	Right Parietal	F
PT245	Glioblastoma, IDHwt, WHO Grade 4	Recurrent	1	Right Frontal	F
PT248	Glioblastoma, IDHwt, WHO Grade 4	Primary	0	Right Frontal	F
PT265	Glioblastoma, IDHwt, WHO Grade 4	Primary	0	Right Temporal	M
PT266	Astrocytoma, IDHmt, WHO Grade 3	Primary	0	Right Temporal	F
PT268	Astrocytoma, IDHwt, WHO Grade 3	Primary	0	Left Parietal	F
PT277	Glioblastoma, IDHwt, WHO Grade 4	Recurrent	3	Right Parietal	F
PT280	Astrocytoma, IDHmt, WHO Grade 2	Primary	0	Right Temporal	M
PT287	Anaplastic Oligodendroglioma, WHO Grade 3	Primary	0	N/A	N/A
PT304	Glioblastoma, IDHwt, WHO Grade 4	Recurrent	3	Left Occipital	F
PT309	Glioblastoma, IDHwt, WHO Grade 4	Primary	0	Left Parietal	M
PT327	Astrocytoma, IDHmt, WHO Grade 4	Recurrent	2	Right Temporal	M
**Matched Patient Tumour & Gliomaspheres**					
PT/GS147	Glioblastoma, IDHwt, WHO Grade 4	Recurrent	2	Right Parietal	M
PT/GS208	Glioblastoma, IDHwt, WHO Grade 4	Primary	0	Right Temporal	M
PT/GS258	Glioblastoma, IDHwt, WHO Grade 4	Primary	0	Right Temporal	M
**Gliomaspheres**					
GBM39	Glioblastoma, IDHwt, WHO Grade 4	Primary	0	Frontal	M
GS001	Glioblastoma, IDHwt, WHO Grade 4	Primary	0	Right Parietal	M
GS005	Glioblastoma, IDHwt, WHO Grade 4	Recurrent	2	Left Temporal	M
GS024	Glioblastoma, IDHwt, WHO Grade 4	Primary	0	Right Temporal	F
GS025	Glioblastoma, IDHwt, WHO Grade 4	Recurrent	4	Left Frontal	F
GS026	Oligodendroglioma, IDHmt, 1p19q_codel, WHO Grade 3	Recurrent	10	Right Frontal	M
GS027	Glioblastoma, IDHwt, WHO Grade 4	Recurrent	3	Right Parietal	F
GS028	Glioblastoma, IDHwt, WHO Grade 4	Recurrent	1	Right Parietal	F
GS054	Glioblastoma, IDHwt, WHO Grade 4	Recurrent	1	Right Temporal	F
GS075	Glioblastoma, IDHwt, WHO Grade 4	Primary	0	Left Frontal	F
GS081	Glioblastoma, IDHwt, WHO Grade 4	Primary	0	Left Frontal	F
GS090	Glioblastoma, IDHwt, WHO Grade 4	Recurrent	1	Right Parietal	M
GS100	Glioblastoma, IDHwt, WHO Grade 4	Recurrent	2	Right Temporal	M
GS104	Glioblastoma, IDHwt, WHO Grade 4	Primary	0	Left Parietal	M
GS108	Astrocytoma, IDHmt, WHO Grade 4	Recurrent	1	Rgiht Occipital	M
GS114	Glioblastoma, IDHwt, WHO Grade 4	Primary	0	Right Temporal	M
GS116	Glioblastoma, IDHwt, WHO Grade 4	Primary	0	Left Frontal	M
GS118	Glioblastoma, IDHwt, WHO Grade 4	Recurrent	1	Right Temporal	M
GS121	Glioblastoma, IDHwt, WHO Grade 4	Primary	0	Left Temporal	M
GS122	Glioblastoma, IDHwt, WHO Grade 4	Recurrent	2	Right Temporal	M
GS187	Glioblastoma, IDHwt, WHO Grade 4	Primary	0	Right Temporal	F
HK301	Glioblastoma, IDHwt, WHO Grade 4	Primary	0	Left Parietal	M
HK336	Glioblastoma, IDHwt, WHO Grade 4	Primary	0	Left Frontal	M

Supplemental Table 1: Patient and Model Characteristics

Final diagnosis, tumour status, tumour location and sex for all glioma samples and derivative models in Figure 1. Headings are in bold text.

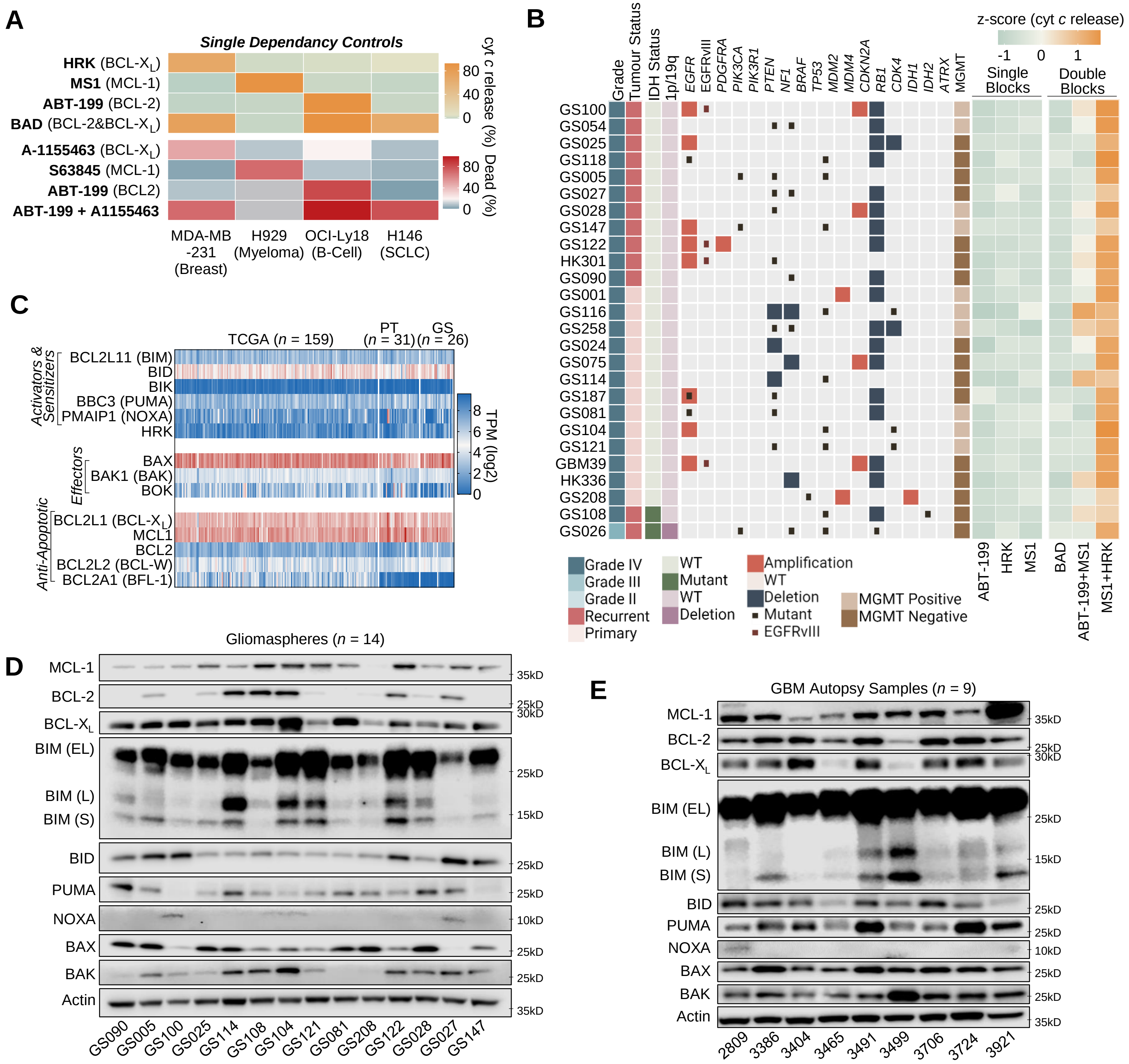
A
Single Dependancy Controls
HRK (BCL-XL)
MS1 (MCL-1)
ABT-199 (BCL-2)
BAD (BCL-2&BCL-XL)
A-1155463 (BCL-XL)
S63845 (MCL-1)
ABT-199 (BCL2)
ABT-199 + A1155463
cyt c release (%)
Dead (%)
MDA-MB -231 (Breast)
H929 (Myeloma)
OCI-Ly18 (B-Cell)
H146 (SCLC)
B
z-score (cyt c release)
Single Blocks
Double Blocks
Grade
Tumour Status
IDH Status
1p/19q
EGFR
EGFRvIII
PDGFRA
PIK3CA
PIK3R1
PTEN
NF1
BRAF
TP53
MDM2
MDM4
CDKN2A
RB1
CDK4
IDH1
IDH2
ATRX
MGMT
GS100
GS054
GS025
GS118
GS005
GS027
GS028
GS147
GS122
HK301
GS090
GS001
GS116
GS258
GS024
GS075
GS114
GS187
GS081
GS104
GS121
GBM39
HK336
GS208
GS108
GS026
ABT-199
HRK
MS1
BAD
ABT-199+MS1
MS1+HRK
Grade IV
Grade III
Grade II
Recurrent
Primary
WT
Mutant
WT
Deletion
Amplification
WT
Deletion
Mutant
EGFRvIII
MGMT Positive
MGMT Negative
C
TCGA (n = 159)
PT (n = 31)
GS (n = 26)
Activators & Sensitizers
BCL2L11 (BIM)
BID
BIK
BBC3 (PUMA)
PMAIP1 (NOXA)
HRK
Effectors
BAX
BAK1 (BAK)
BOK
Anti-Apoptotic
BCL2L1 (BCL-XL)
MCL1
BCL2
BCL2L2 (BCL-W)
BCL2A1 (BFL-1)
TPM (log2)
D
Gliomaspheres (n = 14)
MCL-1
BCL-2
BCL-XL
BIM (EL)
BIM (L)
BIM (S)
BID
PUMA
NOXA
BAX
BAK
Actin
35kD
25kD
30kD
15kD
10kD
GS090
GS005
GS100
GS025
GS114
GS108
GS104
GS121
GS081
GS208
GS122
GS028
GS027
GS147
E
GBM Autopsy Samples (n = 9)
MCL-1
BCL-2
BCL-XL
BIM (EL)
BIM (L)
BIM (S)
BID
PUMA
NOXA
BAX
BAK
Actin
2809
3386
3404
3465
3491
3499
3706
3724
3921

Supplemental Figure 1: Molecular and functional characterization of the intrinsic apoptotic machinery in GBM identifies survival essential dependence on BCLXL and MCL1

A. BH3 profiling of anti-apoptotic blocks in non-GBM cancer cell lines. Peptide concentrations are as follows: ABT-199: 1µM, MS1: 10µM, HRK: 100µM, BAD: 10µM. Lower heatmap of cell viability (Cell Titer Glo) after 48 hrs. of treatment with 0.5µM ABT-199 (BCL2i), 0.5µM A1155463 (BCL-X_Li), and 0.5µM S63856 (MCL1i).

B. Heat maps describing gliomasphere clinical characteristics, copy number alterations and mutations and BH3 profiling of the apoptotic blocks. BH3 profiling is plotted as a z-score across the sample. Peptide concentrations are as follows: ABT-199: 1µM, MS1: 10µM, HRK: 100µM, BAD: 10µM.

C. Assessment of RNA expression of the BCL2 protein family from GBM tumour cohort (TCGA, *n* = 159), patient tumours (*n* = 31) and gliomaspheres (*n* = 26). Expression values in transcripts per million (TPM), log2 transformed.

D. Immunoblot analysis of the intrinsic apoptotic machinery in gliomaspheres (*n* = 13).

E. Immunoblot analysis of intrinsic apoptotic machinery in GBM autopsy samples (*n* = 9) obtained from UCLA pathology.

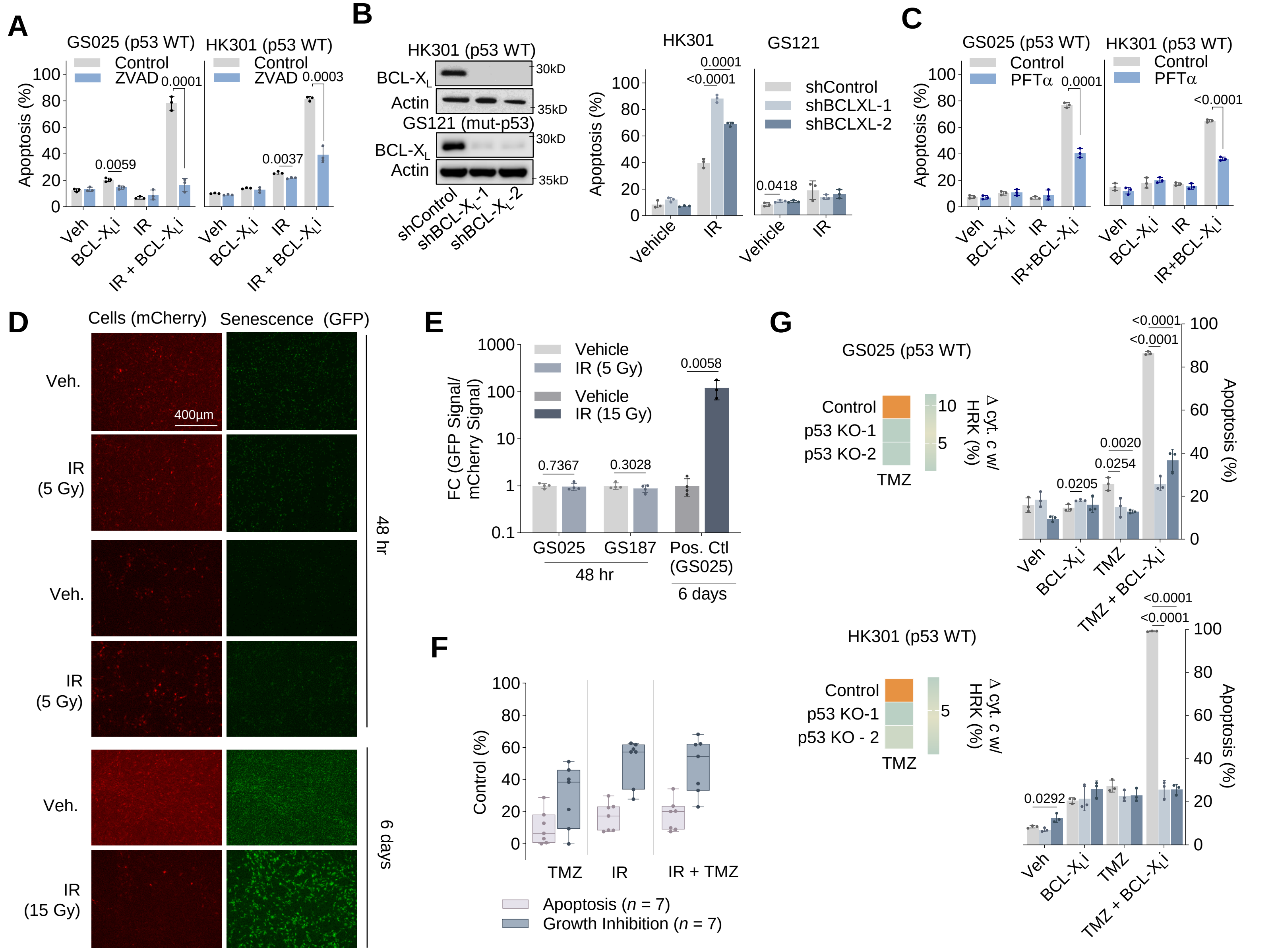

Supplemental Figure 2: IR creates an exclusive survival dependency on BCL-X_L in p53 wild-type GBM

A. Apoptosis measured post treatment with BCL-X_li, IR, or IR + BCL-X_Li. All condition were also combined with the pan caspase inhibitor ZVAD-fmk (50µM) (mean ± s.d., unpaired t test with Welch correction, *n* = 3 biological replicates).

B. Short-hairpin RNAs were used reduce expression of BCL-X_L. Apoptosis assessed after treatment with IR (mean ± s.d., unpaired t test with Welch correction, *n* = 3 biological replicates).

C. Apoptosis was measured post treatment with BCL-X_li, IR, or IR + BCL-X_Li. All condition were also combined with the p53 transcriptional inhibitor, PFTα (mean ± s.d., unpaired t test with Welch correction, *n* = 3 biological replicates).

D. Representative images of senescence staining, 48 hours post treatment with 5 Gy IR. Positive control taken 16 days post treatment with 15 Gy IR. Images taken on EVOS M5000.

E. Quantified signal of senescence staining, 48 hours post treatment with 5 Gy IR. Positive control taken 16 days post treatment with 15 Gy IR. Quantification perform on Incucyte (mean ± s.d., unpaired t test with Welch correction, *n* = 4 biological replicates). These results were independently repeated.

F. Box plots of growth inhibition and apoptosis in patient-derived gliomaspheres (*n* = 7) treated with IR, TMZ or IR + TMZ. Each dot represents an individual gliomasphere and the mean of three biological replicates (mean ± s.d., unpaired t test with Welch correction).

G. Dynamic BH3 profiling of p53 KOs after TMZ treatment, shows change in precent cytochrome *c* release with HRK (mean ± s.d.). Cell death was evaluated in p53 KOs, 5 days after treatment with BCL-X_Li, TMZ , or TMZ+ BCL-X_Li, (mean ± s.d., unpaired t test with Welch correction, *n* = 2 biological replicates for Dynamic BH3 profiling, n = 3 biological replicates for Annexin V/PI+).

IR (5gy), TMZ (50µM), BCL-X_Li (A1155463: 0.5µM).
DBP Assessed at 48 hours, peptide concentrations: HRK: 100µM.
Apoptosis (Annexin V/PI) assessed at 5 days. All box plots: mean, hinges at 25th and 75th percentiles, ± min to max

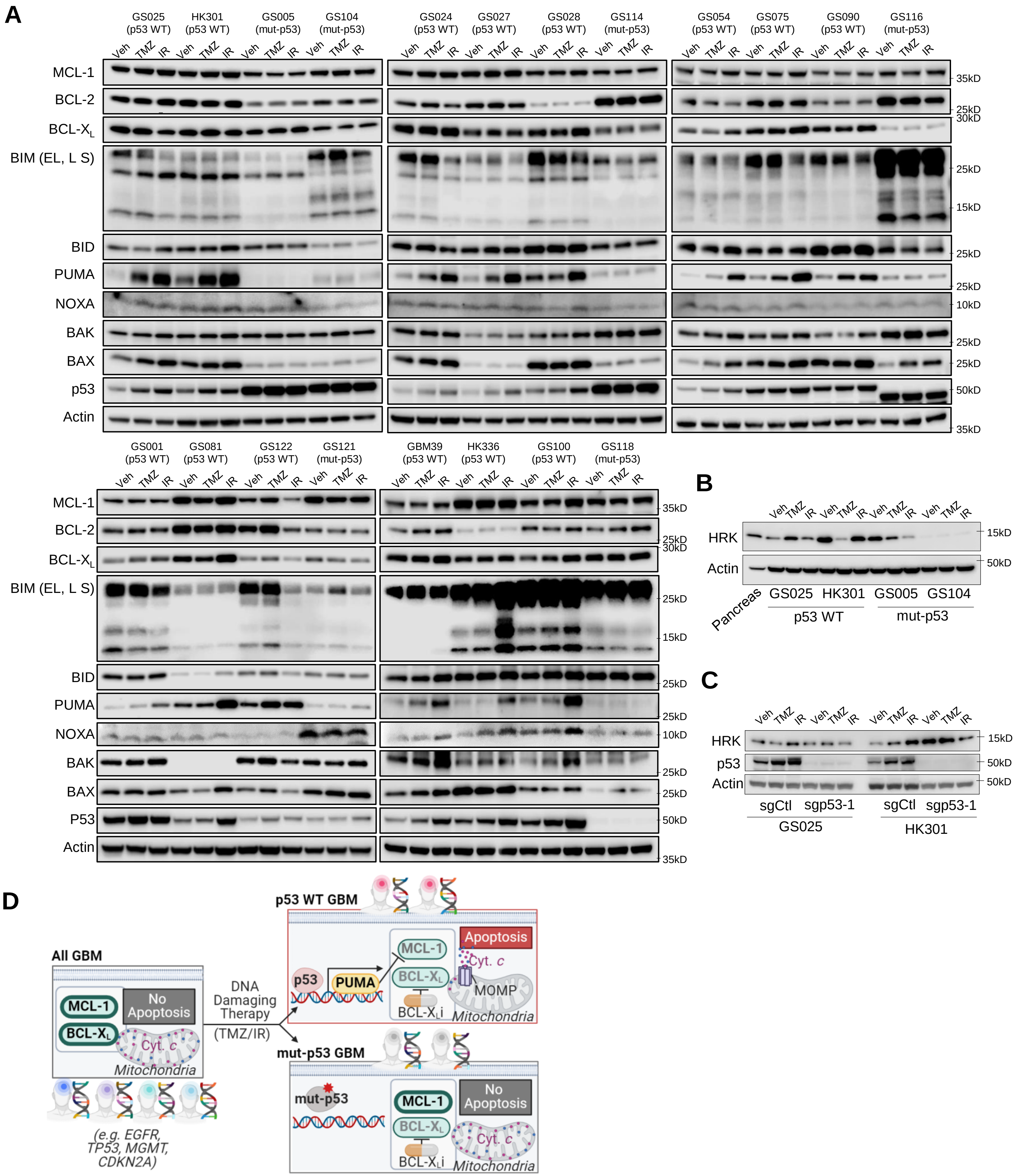
A
GS025 (p53 WT)
HK301 (p53 WT)
GS005 (mut-p53)
GS104 (mut-p53)
GS024 (p53 WT)
GS027 (p53 WT)
GS028 (p53 WT)
GS114 (mut-p53)
GS054 (p53 WT)
GS075 (p53 WT)
GS090 (p53 WT)
GS116 (mut-p53)
Veh
TMZ
IR
MCL-1
BCL-2
BCL-XL
BIM (EL, L S)
BID
PUMA
NOXA
BAK
BAX
p53
Actin
35kD
25kD
30kD
15kD
10kD
50kD
GS001 (p53 WT)
GS081 (p53 WT)
GS122 (p53 WT)
GS121 (mut-p53)
GBM39 (p53 WT)
HK336 (p53 WT)
GS100 (p53 WT)
GS118 (mut-p53)
P53
B
HRK
Actin
Pancreas
GS025
HK301
GS005
GS104
p53 WT
mut-p53
C
sgCtl
sgp53-1
GS025
HK301
D
All GBM
MCL-1
BCL-XL
No Apoptosis
Cyt. c
Mitochondria
(e.g. EGFR, TP53, MGMT, CDKN2A)
DNA Damaging Therapy (TMZ/IR)
p53 WT GBM
p53
PUMA
BCL-XLi
Apoptosis
MOMP
mut-p53 GBM
mut-p53

Supplemental Figure 3: TMZ/IR upregulation of PUMA neutralizes MCL1 to create a BCLXL dependency

A. Immunoblots to assess changes in the expression of BCL2 family proteins 48 hours post TMZ (50µM) or IR (5 Gy) treatment across gliomaspheres (*n* = 19).

B. Immunoblots to assess changes in the expression of HRK 48 hours post TMZ (50µM) or IR (5 Gy) treatment across gliomaspheres (*n* = 4).

C. Immunoblots to assess changes in the expression of HRK 48 hours post TMZ (50µM) or IR (5 Gy) treatment in p53 KO gliomaspheres, GS025 and HK301.

D. Graphic summary of the apoptotic blocks and their response to standard of care treatments in GBM.

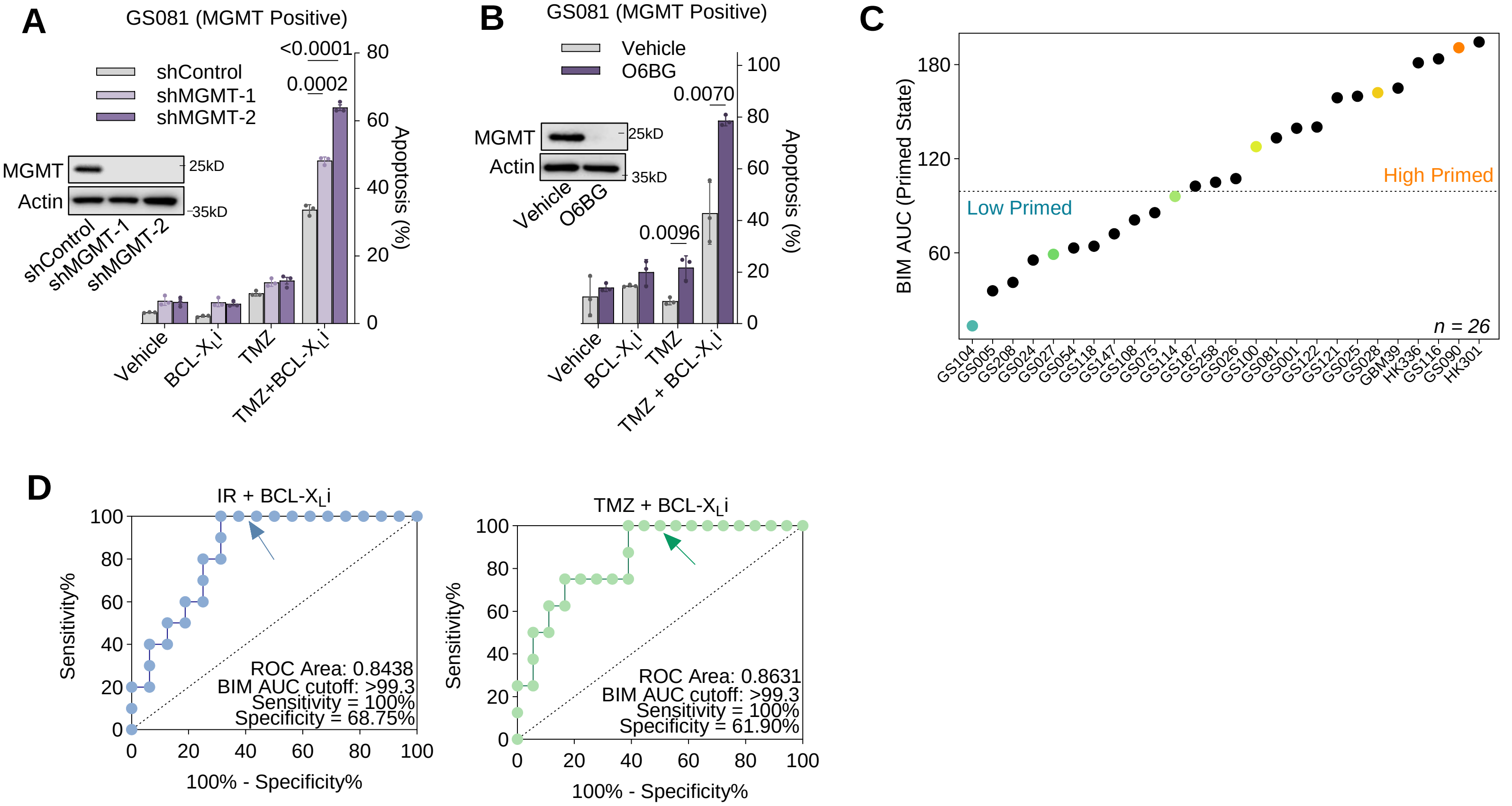

Supplemental Figure 4: p53 status alone cannot predict response to IR/TMZ in combination with BCL-X_Li

A. Short-hairpin RNAs were used reduce expression MGMT in MGMT positive, GS081. Immunoblots show expression of MGMT and Actin in GS081-shControl, shMGMT-1 and shMGMT-2. Apoptosis (Annexin V/PI+) was evaluated in GS081-shControl, shMGMT-1 and shMGMT-2, 5 days after treatment with A1155463 (BCL-X_Li – 0.5µM), TMZ (50µM) or TMZ+A1155463 (mean ± s.d., unpaired t test with Welch correction, *n* = 3 biological replicates).

B. Apoptosis was also evaluated with the listed treatments in panel A, combined with the irreversible MGMT inhibitor O6BG (40µM) (mean ± s.d., unpaired t test with Welch correction, *n* = 3 biological replicates).

C. Basal BH3 profiling of gliomaspheres (*n* = 26) preformed with a titration of the BIM peptide (0µM, 0.01µM, 0.03µM, 0.1µM, 0.3µM, 1µM, 3µM, 10µM). Curve in the dynamic range (0.03µM - 3µM BIM) used to calculate area under the curve (AUC) to describe the primed state of these tumours. Colored dots match the colored curves in Figure 3C.

D. Receiver Operator Curves (ROC) used to determine the priming cut-off where BIM^{AUC} has the most sensitive prediction of cell death. Cut-off used to draw the line in panel C.

p > 0.05 = ns; p < 0.05 = *; p < 0.01 = **; p < 0.001 = ***; p < 0.0001 = ****

Supplemental Table 2 - Model Descriptions	
Abbreviation	Full formula
IMF	BIMAUC*TP53 mutation*MGMT status*MDM2 amplification (p = 15)
GM	RNA expression (p = 18,909) + Copy Number Alterations (p = 19,023) + Mutation calls (p = 213)
GM(*)	Top RNA1*Top RNA2*…*Top CNA1* top CNA2*…*Top MUT1*top MUT2… Up to three-way interactions between all top features in RNA, CNA and MUT. Selected by highest absolute Pearson correlations with the outcome IR/TMZ + BCLXLi-induced apoptosis. (p = 10,995 combinations)
IMF + GM	BIMAUC*TP53 mutation*MGMT status*MDM2 amplification+ RNA+CNA+MUT (p = 15 + 18,909 + 19,023+ 213)

Supplemental Table 2: Model Descriptions

Abbreviations and full formulas for the machine learning models.

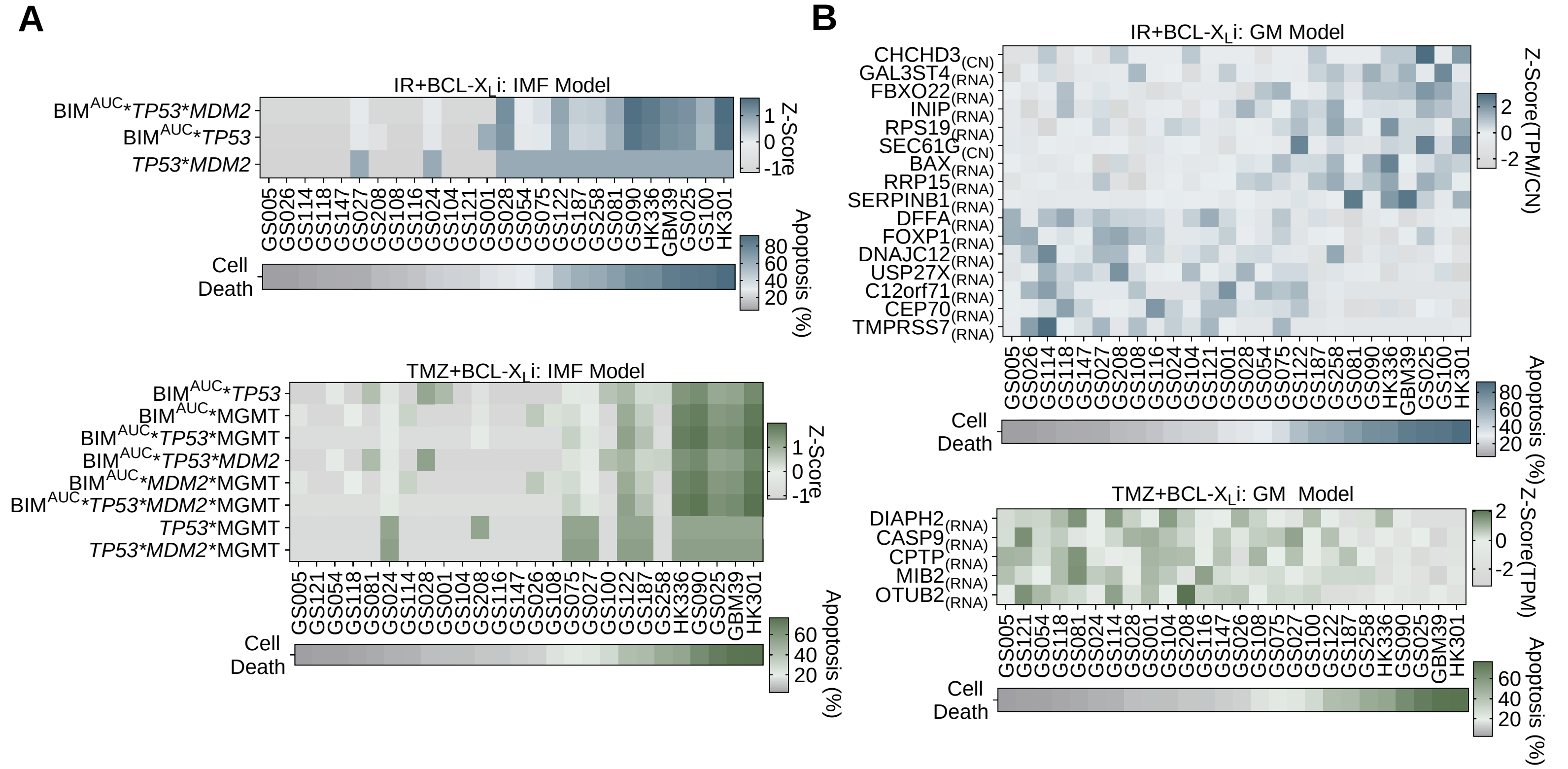

C

Feature	Description
**IR + BCL-X_Li ~ GM Features Descriptions**	
CHCHD3$_{(CN)}$	Inner mitochondrial membrane scaffold protein
GAL3ST4$_{(RNA)}$	Galactose-3-O-sulfotransferase protein family
FBXO22$_{(RNA)}$	Member of the F-box protein, transcriptional target of the tumour protein p53
INIP$_{(RNA)}$	Subunit of single-stranded DNA binding complexes, maintains genome stability
RPS19$_{(RNA)}$	Ribosomal protein that is a component of the 40S subunit
SEC61G$_{(CN)}$	Component of the protein translocation apparatus of the endoplasmic reticulum
BAX$_{(RNA)}$	BCL2 protein family, functions as an apoptotic activator
RRP15$_{(RNA)}$	Protein that co-purifies with human nucleoli
SERPINB1$_{(RNA)}$	Member of the serpin family of proteinase inhibitors
DFFA$_{(RNA)}$	Substrate for caspase-3 and triggers DNA fragmentation during apoptosis
FOXP1$_{(RNA)}$	Tissue- and cell type-specific gene transcription, tumour sup.
DNAJC12$_{(RNA)}$	Subclass of the HSP40/DnaJ protein family
USP27X$_{(RNA)}$	Deubiquitinase that is involved in upregulation of the pro-apoptotic Bim protein
C12orf71$_{(RNA)}$	Chromosome 12 open reading frame 71
CEP70$_{(RNA)}$	Enables identical protein binding activity
TMPRSS7$_{(RNA)}$	Predicted to enable serine-type peptidase activity
**TMZ + BCL-X_Li ~ GM Features Descriptions**	
Feature	Description
DIAPH2(RNA)	Diaphanous subfamily of the formin homology family of proteins
CASP9(RNA)	Cysteine-aspartic acid protease (caspase) family, execution-phase of cell apoptosis
CPTP(RNA)	Enables ceramide 1-phosphate binding and ceramide 1-phosphate transfer activity
MIB2(RNA)	E3 ubiquitin protein ligase that mediates ubiquitination in the Notch signaling pathway
OTUB2(RNA)	Deubiquitylating enzymne

Supplemental Figure 5: Feature correlation and description for features chosen at least 10/26 rounds of LOOCV for IMF and GM models.

A. Z-score of the individual features selected for IMF models describing IR/TMZ + BCL-X_L cell death. The heatmap title formula is a high-level description before expanding to all possible combinations of interactions.

B. Gene expression values for selected features by elastic net for the GM models describing IR/TMZ + BCL-XL cell death. Features displayed chosen at least 10/26 rounds of LOOCV. Z-score of log2 transformed counts per million.

C. Selected features by elastic net for GM models describing IR/TMZ + BCL-X_L cell death with description summarizing gene function (paraphrased NCBI databases) displayed. Headings are in bold text.

Features pulled from RNA data set labeled as $_{(RNA)}$, features pulled from copy number data set labeled as $_{(CN)}$.

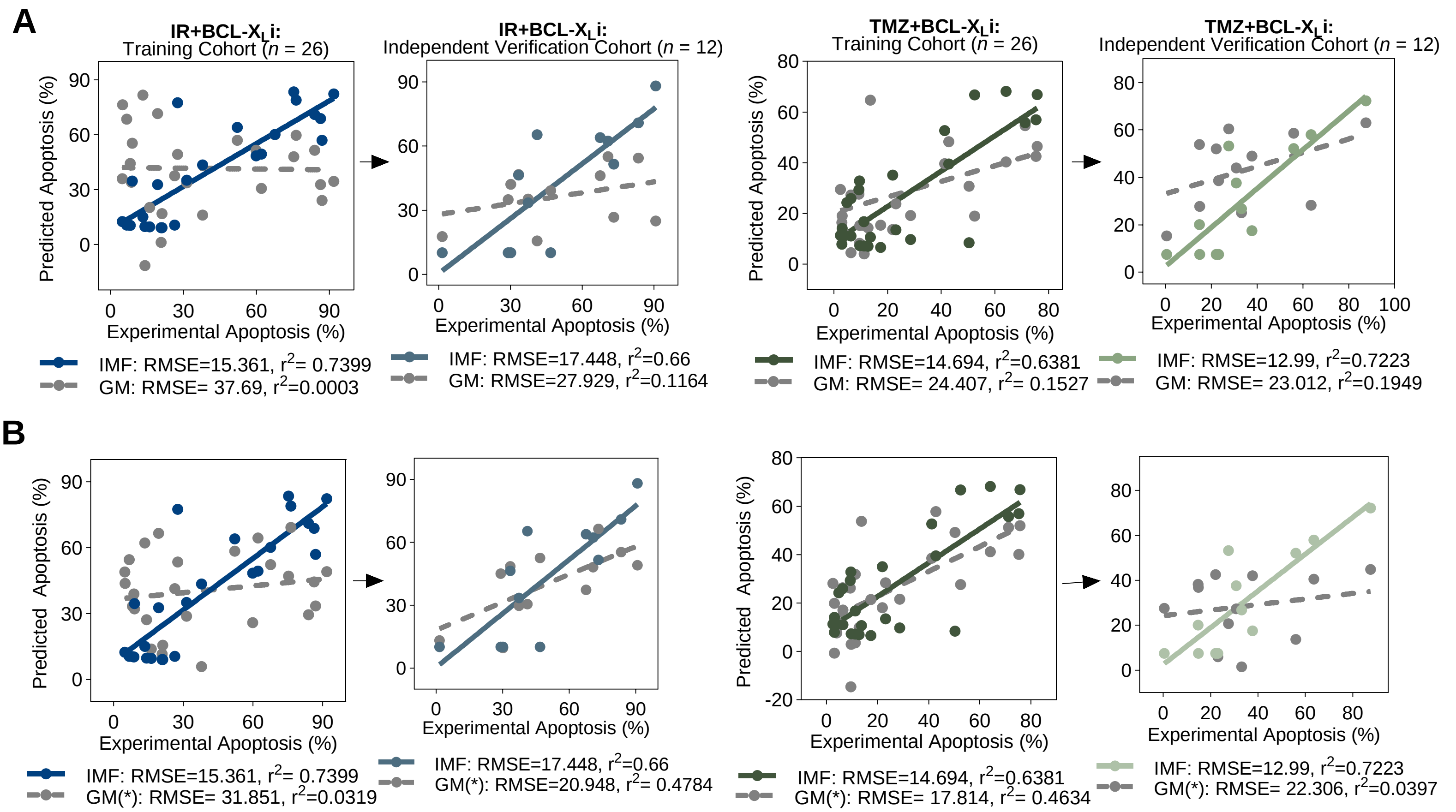

C

**IR + BCL-X_Li ~ IMF + GM**		**TMZ + BCL-X_Li ~ IMF + GM**	
permuation p value: 0.009		permuation p value: 0.018	
feature	order	feature	order
BIM**TP53*MDM2*	1	BIM**TP53*MDM2**MGMT	1
BIM**TP53*	2	CASP9$_{(RNA)}$	2
CASP9$_{(RNA)}$	4	SCML1$_{(RNA)}$	4
CCKBR$_{(RNA)}$	4	SH2D7$_{(RNA)}$	4
SNAP23$_{(RNA)}$	5	GUK1$_{(RNA)}$	5
FBXO22$_{(RNA)}$	8	COBLL1$_{(RNA)}$	6
RNF225$_{(RNA)}$	8	RAB3B$_{(RNA)}$	8
SEC61G$_{(CN)}$	8	RAB43$_{(RNA)}$	8
ADGRG5$_{(RNA)}$	11	TCAF1$_{(RNA)}$	9
KRTAP19.8$_{(RNA)}$	11	PRADC1$_{(RNA)}$	11
CHCHD3$_{(CN)}$	11	TM4SF20$_{(RNA)}$	11

Supplemental Figure 6: Integrated profiling outperforms global transcriptomic and genomic data sets

A. Comparison of experimental and predicted values via simple linear regression for the IMF and GM models
B. Comparison of experimental and predicted values via simple linear regression for the IMF and GM(*) models
C. Rank of pooled IMF + GM features based on retention in LASSO with increasing penalization.

Features pulled from RNA data set labeled as $_{(RNA)}$, features pulled from copy number data set labeled as $_{(CN)}$.

Supplemental Table 3: Patient Characteristics - Independent Verficiation Cohort					
**Gliomaspheres**	**Final Diagnosis**	**Tumour Status**	**Recurrence Number**	**Lobe**	**Sex**
GS055	Glioblastoma, IDHwt, WHO Grade 4	Recurrent	unknown	Left Temporal	M
GS074	Glioblastoma, IDHwt, WHO Grade 4	Primary	0	Left Temporal	M
GS158	Glioblastoma, IDHwt, WHO Grade 4	Recurrent	3	Left Occipital	M
GS176	Glioblastoma, IDHwt, WHO Grade 4	Primary	0	Left Temporal	F
GS180	Glioblastoma, IDHwt, WHO Grade 4	Recurrent	1	Left Temporal	F
GS220	Glioblastoma, IDHwt, WHO Grade 4	Primary	0	Left Temporal	F
GS227	Glioblastoma, IDHwt, WHO Grade 4	Primary	0	Right Parietal	F
GS243	Glioblastoma, IDHwt, WHO Grade 4	Primary	0	Right Temporal	M
GS248	Glioblastoma, IDHwt, WHO Grade 4	Primary	0	Right Frontal	F
GS304	Glioblastoma, IDHwt, WHO Grade 4	Recurrent	2	Left Occipital	F
GS306	Glioblastoma, IDHwt, WHO Grade 4	Recurrent	1	Left Parietal	F
GS319	Glioblastoma, IDHwt, WHO Grade 4	Primary	0	Right Parietal	M

Supplemental Table 3: Patient Characteristics

Final diagnosis, tumour status, tumour location and sex for the glioma samples used to derive the gliomaspheres in the validation cohort. Headings are in bold text.

Supplemental Table 4: Features for IMF, GM and GM(*) models

Feature	Coef.	SD	Stand. Coef.
IR + BCL-X$_L$i ~ IMF Model			
(Intercept)	10.2		
BIM^{AUC}*TP53	0.11	72.09	8.15
BIM^{AUC}*TP53*MDM2	0.26	73.97	19.57
TMZ + BCL-X$_L$i ~ IMF Model			
(Intercept)	7.48		
BIM^{AUC}*TP53*MDM2	0.09	73.97	6.74
BIM^{AUC}*TP53*MGMT	0.1	72.92	7.18
BIM^{AUC}*TP53*MDM2*MGMT	0.12	73.63	9.17
IR + BCL-XLi ~ GM Model			
(Intercept)	-125.57		
CHCHD3$_{(CN)}$	8.82	0.38	3.38
GAL3ST4$_{(RNA)}$	8.30	1.05	8.69
FBXO22$_{(RNA)}$	8.14	0.61	4.96
INIP$_{(RNA)}$	7.40	0.42	3.08
RPS19$_{(RNA)}$	5.75	0.44	2.52
SEC61G$_{(CN)}$	4.53	1.30	5.90
BAX$_{(RNA)}$	3.80	0.78	2.98
RRP15$_{(RNA)}$	3.46	0.47	1.62
SERPINB1$_{(RNA)}$	2.16	1.51	3.26
DFFA$_{(RNA)}$	-0.99	0.58	-0.57
FOXP1$_{(RNA)}$	-1.16	1.50	-1.75
DNAJC12$_{(RNA)}$	-2.26	1.18	-2.66
USP27X$_{(RNA)}$	-4.18	0.47	-1.95
C12orf71$_{(RNA)}$	-7.07	0.04	-0.26
CEP70$_{(RNA)}$	-7.23	0.70	-5.05
TMPRSS7$_{(RNA)}$	-45.81	0.06	-2.55
TMZ + BCL-XLi: GM Model			
(Intercept)	184.26		
DIAPH2$_{(RNA)}$	-4.14	1.01	-4.18
CASP9$_{(RNA)}$	-7.38	0.70	-5.17
CPTP$_{(RNA)}$	-7.74	0.57	-4.42
MIB2$_{(RNA)}$	-11.16	0.77	-8.55
OTUB2$_{(RNA)}$	-22.64	0.43	-9.62
IR + BCL-XLi ~ GM(*) Model			
(Intercept)	12.16		
DDB2$_{(RNA)}$*NHEJ1$_{(RNA)}$*RNF19A$_{(RNA)}$	0.38	28.01	10.61
BAX$_{(RNA)}$*NHEJ1$_{(RNA)}$*RNF19A$_{(RNA)}$	0.14	34.50	4.90
CASP9$_{(RNA)}$*FOXP1$_{(RNA)}$*SCML1$_{(RNA)}$	-0.57	24.72	-14.10
TMZ + BCL-XLi ~ GM(*) Model			
(Intercept)	57.55		
CASP9$_{(RNA)}$*COBLL1$_{(RNA)}$*MIB2$_{(RNA)}$	-0.12	22.83	-2.67
CASP9$_{(RNA)}$*COBLL1$_{(RNA)}$*PPP1R16A$_{(RNA)}$	-0.18	19.65	-3.49
CASP9$_{(RNA)}$*COBLL1$_{(RNA)}$*STX18$_{(RNA)}$	0.02	17.34	0.40
CASP9$_{(RNA)}$*CPTP$_{(RNA)}$*STX4$_{(RNA)}$	-0.11	24.85	-2.76
CASP9$_{(RNA)}$*DIAPH2$_{(RNA)}$*OTUB2$_{(RNA)}$	-0.52	6.45	-3.35
CASP9$_{(RNA)}$*STX18$_{(RNA)}$*STX4$_{(RNA)}$	-0.09	20.87	-1.91
CCDC96$_{(RNA)}$*FBXO48$_{(RNA)}$	-0.72	0.53	-0.38
CPTP$_{(RNA)}$*DIAPH2$_{(RNA)}$*OTUB2$_{(RNA)}$	-0.25	8.59	-2.19
CPTP$_{(RNA)}$*FBXO48$_{(RNA)}$	-0.82	1.16	-0.95
CPTP$_{(RNA)}$*OTUB2$_{(RNA)}$	-0.16	2.33	-0.37
CPTP$_{(RNA)}$*STX18$_{(RNA)}$*STX4$_{(RNA)}$	-0.07	22.74	-1.63
INAFM2$_{(RNA)}$*NHEJ1$_{(RNA)}$	0.94	3.47	3.28
INAFM2$_{(RNA)}$*NHEJ1$_{(RNA)}$*CHCHD3$_{(CN)}$	0.96	6.77	6.51

Supplemental Table 4: Features for the IMF, GM and GM(*) models

Features for the IMF, GM and GM(*) models describing IR/TMZ + BCL-X$_L$ cell death with the final model intercepts and coefficient. Standard deviation (SD) and standardized coefficient are displayed to describe the contribution of each feature. Headings are in bold text.

A

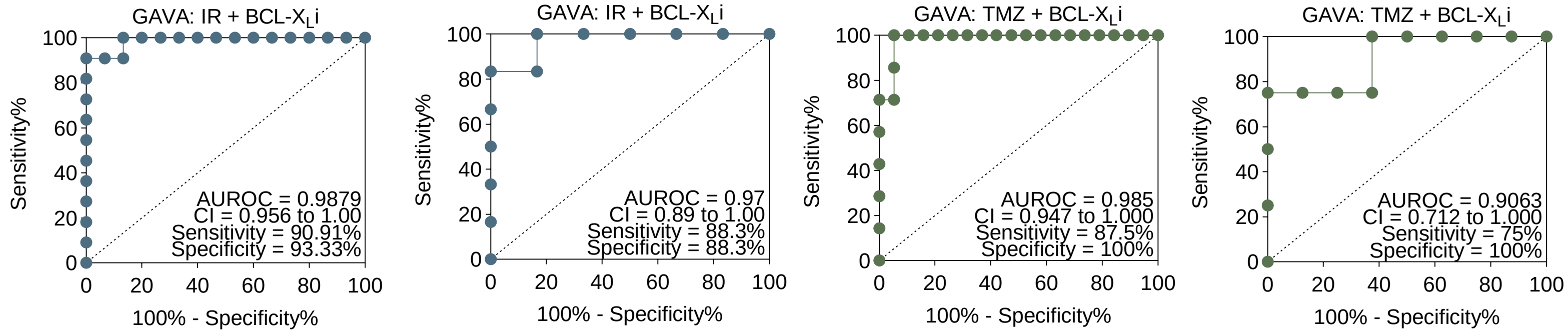

Supplemental Figure 7: Evaluation of IMF as a binary biomarker.

A. ROC curves used to evaluate the GAVA model as a binary score.

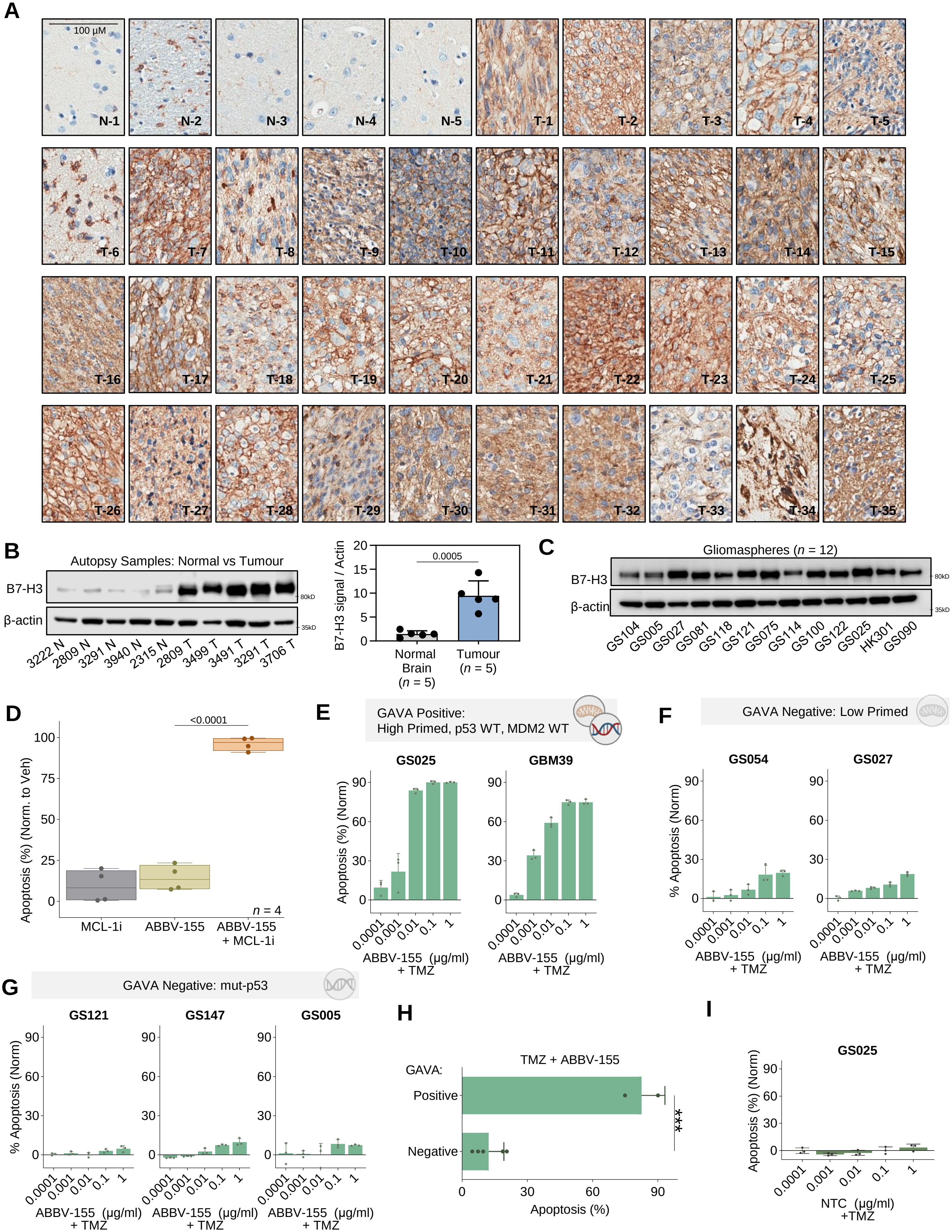
A
100 µM
N-1
N-2
N-3
N-4
N-5
T-1
T-2
T-3
T-4
T-5
T-6
T-7
T-8
T-9
T-10
T-11
T-12
T-13
T-14
T-15
T-16
T-17
T-18
T-19
T-20
T-21
T-22
T-23
T-24
T-25
T-26
T-27
T-28
T-29
T-30
T-31
T-32
T-33
T-34
T-35
B
Autopsy Samples: Normal vs Tumour
B7-H3
β-actin
80kD
35kD
3222 N
2809 N
3291 N
3940 N
2315 N
2809 T
3499 T
3491 T
3291 T
3706 T
B7-H3 signal / Actin
0.0005
Normal Brain (n = 5)
Tumour (n = 5)
C
Gliomaspheres (n = 12)
B7-H3
β-actin
GS104
GS005
GS027
GS081
GS118
GS121
GS075
GS114
GS100
GS122
GS025
HK301
GS090
D
Apoptosis (%) (Norm. to Veh)
<0.0001
n = 4
MCL-1i
ABBV-155
ABBV-155 + MCL-1i
E
GAVA Positive:
High Primed, p53 WT, MDM2 WT
GS025
GBM39
Apoptosis (%) (Norm)
ABBV-155 (µg/ml) + TMZ
F
GAVA Negative: Low Primed
GS054
GS027
% Apoptosis (Norm)
ABBV-155 (µg/ml) + TMZ
G
GAVA Negative: mut-p53
GS121
GS147
GS005
% Apoptosis (Norm)
ABBV-155 (µg/ml) + TMZ
H
TMZ + ABBV-155
GAVA:
Positive
Negative
***
Apoptosis (%)
I
GS025
Apoptosis (%) (Norm)
NTC (µg/ml) +TMZ

Supplemental Fig. 8. Novel anti-body drug conjugate provides therapeutically safe alternative to targeting BCL-X_L and is brain penetrant

A. Representative images from immunohistochemistry staining for B7-H3 (CD276) in GBM (*n* = 34) and normal brain (*n* = 5) microarray.

B. Immunoblot and quantification of B7-H3 expression from normal brain (mean ± s.d., *n* = 5) and autopsy samples (mean ± s.d., *n* = 5). Comparisons were made with two-tailed, unpaired t tests.

C. Immunoblot of B7-H3 expression in gliomaspheres (*n* = 12).

D. Apoptosis (Annexin V/PI) of p53 WT (*n* = 2) and mut-p53 (*n* = 2) gliomaspheres treated with 0.5µM MCL-1i (S63845) and 1µg/mL ABBV-155 for 5 days. Data are normalized to vehicle (mean ± s.d., two-tailed, paired t test).

E. Apoptosis (Annexin V/PI +) of GAVA positive, p53 WT and high primed gliomaspheres, GS025 and GBM39, 5 days post TMZ (50uM) and ABBV-155 titration. Concentration range: 0.0001µg/mL, 0.001µg/mL, 0.01µg/mL, 0.1µg/mL, 1µg/mL. Data are normalized to R alone (mean ± s.d., *n* = 3 biological replicates).

F. Apoptosis (Annexin V/PI +) of GAVA negative, low primed gliomaspheres, GS054 and GS027, 5 days post TMZ (50uM) and ABBV-155 titration. Concentration range: 0.0001µg/mL, 0.001µg/mL, 0.01µg/mL, 0.1µg/mL, 1µg/mL. Data are normalized to IR alone (mean ± s.d., *n* = 3 biological replicates).

G. Apoptosis (Annexin V/PI +) GAVA negative, mut-p53 gliomaspheres, GS121, GS147 and GS005, 5 days post TMZ (50uM) and ABBV-155 titration. Concentration range: 0.0001µg/mL, 0.001µg/mL, 0.01µg/mL, 0.1µg/mL, 1µg/mL. Data are normalized to R alone (mean ± s.d., *n* = 3 biological replicates).

H. Grouped analysis by GAVA status of all TMZ + 0.1µg/mL ABBV-155 cell death data (mean ± s.d., student's two sample unpaired t test, *n* = 7 gliomaspheres).

I. Apoptosis (Annexin V/PI +) of p53 WT gliomaspheres, GS025, 5 days post IR (5 Gy) or TMZ (50µM) and Non-Targeting Control (NTC). Concentration range: 0.0001µg/mL, 0.001µg/mL, 0.01µg/mL, 0.1µg/mL, 1µg/mL. Data are normalized to either TMZ or IR alone (mean ± s.d., *n* = 3 biological replicates).
