# Supplementary material for: Integrated molecular and functional characterization of the intrinsic apoptotic machinery identifies therapeutic vulnerabilities in glioma
Source: Nat Commun. 2024 Nov 21;15:10089. doi: 10.1038/s41467-024-54138-9 (PMC11582606; doi:10.1038/s41467-024-54138-9)
Supplement: Supplementary file 2 — Reporting Summary [file 41467_2024_54138_MOESM2_ESM.pdf]

nature portfolio

Corresponding author(s): David Nathanson

Last updated by author(s): 10/12/2024

Reporting Summary

Nature Portfolio wishes to improve the reproducibility of the work that we publish. This form provides structure for consistency and transparency in reporting. For further information on Nature Portfolio policies, see our Editorial Policies and the Editorial Policy Checklist.

Statistics

For all statistical analyses, confirm that the following items are present in the figure legend, table legend, main text, or Methods section.

n/a	Confirmed	
☐	☒	The exact sample size (*n*) for each experimental group/condition, given as a discrete number and unit of measurement
☐	☒	A statement on whether measurements were taken from distinct samples or whether the same sample was measured repeatedly
☐	☒	The statistical test(s) used AND whether they are one- or two-sided<br>*Only common tests should be described solely by name; describe more complex techniques in the Methods section.*
☒	☐	A description of all covariates tested
☒	☐	A description of any assumptions or corrections, such as tests of normality and adjustment for multiple comparisons
☐	☒	A full description of the statistical parameters including central tendency (e.g. means) or other basic estimates (e.g. regression coefficient) AND variation (e.g. standard deviation) or associated estimates of uncertainty (e.g. confidence intervals)
☐	☒	For null hypothesis testing, the test statistic (e.g. *F*, *t*, *r*) with confidence intervals, effect sizes, degrees of freedom and *P* value noted<br>*Give P values as exact values whenever suitable.*
☒	☐	For Bayesian analysis, information on the choice of priors and Markov chain Monte Carlo settings
☒	☐	For hierarchical and complex designs, identification of the appropriate level for tests and full reporting of outcomes
☐	☒	Estimates of effect sizes (e.g. Cohen's *d*, Pearson's *r*), indicating how they were calculated

Our web collection on statistics for biologists contains articles on many of the points above.

Software and code

Policy information about availability of computer code

Data collection	*Provide a description of all commercial, open source and custom code used to collect the data in this study, specifying the version used OR state that no software was used.*
Data analysis	Statistical analysis were calculated in Prism 10.0, comparisons were made with two-tailed unpaired or paired Student's t tests, and P values <0.05 were considered statistically significant. All data from multiple independent experiments were assumed to be of normal variance. For each experiment, replicates are as noted in the figure legends. Data represent mean ± s.d. values unless otherwise indicated. The code for elastic net and cross validations was written in R (v4.1.2).

For manuscripts utilizing custom algorithms or software that are central to the research but not yet described in published literature, software must be made available to editors and reviewers. We strongly encourage code deposition in a community repository (e.g. GitHub). See the Nature Portfolio guidelines for submitting code & software for further information.

Data

Policy information about availability of data

All manuscripts must include a data availability statement. This statement should provide the following information, where applicable:

- Accession codes, unique identifiers, or web links for publicly available datasets
- A description of any restrictions on data availability
- For clinical datasets or third party data, please ensure that the statement adheres to our policy

The whole exome and RNA data generated in this study have been deposited in the dbGap database under accession code phs003286. Sequencing for samples used

in this paper are marked with variable "FERNANDEZ_2024". The remaining data are available within the Article, Supplementary Information or Source Data file.

Research involving human participants, their data, or biological material

Policy information about studies with human participants or human data. See also policy information about sex, gender (identity/presentation), and sexual orientation and race, ethnicity and racism.

Reporting on sex and gender	Human biospecimens were not chosen based on sex or gender, but were instead chosen based in tissue availability. 28 tumors samples were from females, 29 from males, 1 not reported. Sex was self reported. Findings in this paper are applicable to both sexes.
Reporting on race, ethnicity, or other socially relevant groupings	Race and ethnicity not reported.
Population characteristics	The 58 human glioma samples that were included in this paper includes tumors grades 2, 3 and . Samples included newly diagnosed and recurrent tumors.
Recruitment	Explicit informed consent was obtained from patients.
Ethics oversight	All patient-derived tumor tissue was obtained through the UCLA Institutional Review Board (IRB) protocol 10-000655.

Note that full information on the approval of the study protocol must also be provided in the manuscript.

Field-specific reporting

Please select the one below that is the best fit for your research. If you are not sure, read the appropriate sections before making your selection.

☒ Life sciences ☐ Behavioural & social sciences ☐ Ecological, evolutionary & environmental sciences

For a reference copy of the document with all sections, see nature.com/documents/nr-reporting-summary-flat.pdf

Life sciences study design

All studies must disclose on these points even when the disclosure is negative.

Sample size	No formal statistical method was used to pre-determine sample size. Sample size was determined based on the standard deviation and mean in groups, with an expected alpha of 0.05 and power of 0.8.
Data exclusions	No data were excluded from these studies.
Replication	All data were replicated a minimum of 2 times.
Randomization	Tumour bearing mice were randomized into respective treatment groups.
Blinding	Blinding was not possible due to the nature of the experiments; however, determination of endpoint for mouse-studies was verified by technicians who were unaware of the experimental design/hypothesized outcomes.

Reporting for specific materials, systems and methods

We require information from authors about some types of materials, experimental systems and methods used in many studies. Here, indicate whether each material, system or method listed is relevant to your study. If you are not sure if a list item applies to your research, read the appropriate section before selecting a response.

Materials & experimental systems

n/a	Involved in the study	
☐	☒	Antibodies
☐	☒	Eukaryotic cell lines
☒	☐	Palaeontology and archaeology
☐	☒	Animals and other organisms
☒	☐	Clinical data
☒	☐	Dual use research of concern
☒	☐	Plants

Methods

n/a	Involved in the study	
☒	☐	ChIP-seq
☐	☒	Flow cytometry
☒	☐	MRI-based neuroimaging

Antibodies

Antibodies used	anti-β-actin (8H10D10) mouse mAb (Cell Signaling, 3700), anti-α-tubulin (DM1A) mouse mAb (Cell Signaling, 3873), anti-p53 (DO-1) mouse mAb (Santa Cruz Biotechnology, SC-126), anti-BAX (D2E11) rabbit mAb (Cell Signaling, 5023), anti-BAK rabbit mAb (Cell signaling, 3814), anti-BIM (C34C5) rabbit mAb (Cell Signaling, 2933), anti-BID (Human specific) rabbit mAb (Cell signaling, 2002), anti-PUMA (D30C10) rabbit mAb (Cell Signaling, 12450), anti-Noxa (D8L7U) rabbit mAb (Cell Signaling, 14766), anti-Bcl-2 (50E3) rabbit mAb (Cell Signaling, 2870), anti-BCL-XL (54H6) rabbit mAb (Cell Signaling, 2764), anti-Mcl-1 (D35A5) rabbit mAb (Cell Signaling, 5453), anti-HRK (PRS3771) rabbit mAb, and anti-cytochrome c rabbit mAb (Cell Signaling, 4272), anti-BCL-XL (54H6) rabbit mAb (Cell Signaling, 2764), anti-Mcl-1 (D35A5) rabbit mAb (Cell Signaling, 5453), anti-rabbit IgG HRP-linked (Cell Signaling, 7074) and anti-mouse IgG HRP-linked (Cell Signaling, 7076), B7-H3 (R&D Systems, AF1027).
Validation	All antibodies were purchased from reputable commercial vendors with provided validation information

Eukaryotic cell lines

Policy information about cell lines and Sex and Gender in Research

Cell line source(s)	All cell lines were derived from primary patient tumors obtained at the University of California, Los Angeles. 293FT cells were used for virus generation, and were purchased from Thermo Fisher. All primary GBM cells generated for these studies are available upon request subject to an approved materials transfer agreement (MTA).
Authentication	Authentication of cells was carried out by short-tandem repeat (STR) analysis.
Mycoplasma contamination	Testing was performed every every month and results were negative. Cell lines to be injected into orthotopic xengraft models were tested for mycoplasma contamination immediately prior to injection and were negative.
Commonly misidentified lines (See ICLAC register)	No commonly misidentified cells were used

Animals and other research organisms

Policy information about studies involving animals; ARRIVE guidelines recommended for reporting animal research, and Sex and Gender in Research

Laboratory animals	Female NOD.Cg-Prkdcscid Il2rgtm1Wjl/SzJ mice purchased from the Jackson Laboratory (Strain #:005557) were used for all studies. Mice were injected at approximately 8 weeks of age.
Wild animals	Not used
Reporting on sex	*Indicate if findings apply to only one sex; describe whether sex was considered in study design, methods used for assigning sex. Provide data disaggregated for sex where this information has been collected in the source data as appropriate; provide overall numbers in this Reporting Summary. Please state if this information has not been collected. Report sex-based analyses where performed, justify reasons for lack of sex-based analysis.*
Field-collected samples	Not used
Ethics oversight	All mice were kept under defined pathogen-free conditions at the AAALAC-approved animal facility of the Division of Laboratory Animals (DLAM) at UCLA. All animal experiments were performed with the approval of the UCLA Office of Animal Resource Oversight (OARO).

Note that full information on the approval of the study protocol must also be provided in the manuscript.

Plants

Seed stocks	*Report on the source of all seed stocks or other plant material used. If applicable, state the seed stock centre and catalogue number. If plant specimens were collected from the field, describe the collection location, date and sampling procedures.*
Novel plant genotypes	*Describe the methods by which all novel plant genotypes were produced. This includes those generated by transgenic approaches, gene editing, chemical/radiation-based mutagenesis and hybridization. For transgenic lines, describe the transformation method, the number of independent lines analyzed and the generation upon which experiments were performed. For gene-edited lines, describe the editor used, the endogenous sequence targeted for editing, the targeting guide RNA sequence (if applicable) and how the editor was applied.*
Authentication	*Describe any authentication procedures for each seed stock used or novel genotype generated. Describe any experiments used to assess the effect of a mutation and, where applicable, how potential secondary effects (e.g. second site T-DNA insertions, mosiacism, off-target gene editing) were examined.*

Flow Cytometry

Plots

Confirm that:

- [ ] The axis labels state the marker and fluorochrome used (e.g. CD4-FITC).
- [ ] The axis scales are clearly visible. Include numbers along axes only for bottom left plot of group (a 'group' is an analysis of identical markers).
- [ ] All plots are contour plots with outliers or pseudocolor plots.
- [ ] A numerical value for number of cells or percentage (with statistics) is provided.

Methodology

Sample preparation	Annexin V apoptosis assay: Cells were collected and analyzed for annexin V and PI staining according to the manufacturer's protocol (BD Biosciences). Briefly, cells were plated at 5 × 104 cells/mL and treated with the appropriate drugs. At the indicated time points, cells were collected, trypsinized, washed with PBS, and stained with annexin V and PI for 15 min.<br>BH3 Profiling: Cells were disassociated into single-cell suspensions and resuspended in MEB buffer (150 mM mannitol, 10 mM HEPES-KOH, 50 mM KCl, 0.02 mM EGTA, 0.02 mM EDTA, 0.1% BSA, and 5 mM succinate). 50µL of cell suspension (3 × 104 cells/well) was plated in wells holding 50µL MEB buffer containing 0.002% digitonin and the indicated peptides in 96-well plates. Plates were then incubated at 25 °C for 50 min. Cells were then fixed with 4% paraformaldehyde for 10 min and neutralized with N2 buffer (1.7 M Tris and 1.25 M glycine, pH 9.1) for 5 min. Samples were stained overnight with 20µL of staining solution (10% BSA and 2% Tween 20 in PBS) containing DAPI and anti-cytochrome c (BioLegend clone 6H2.B4, cat. no. 612310)
Instrument	BD LSRII or Attune
Software	FlowJo
Cell population abundance	n/a
Gating strategy	Annexin V apoptosis assay: Total cells were selected for with a forward scatter/side scatter gate, this population was used to examine the Annexin V/PI staining. Vehicle treated cells were used to set gates for comparison to treated cells for analysis of apoptosis.<br>BH3 profiling: After total cells were selected for with a forward scatter/side scatter gate, singlets were gated for DAPI staining to select out sub-G1 (dead) cells. Cytochrome c staining was then analyzed.

- [ ] Tick this box to confirm that a figure exemplifying the gating strategy is provided in the Supplementary Information.
